# Understanding media's leverage in the national elite sport ecosystems

**DOI:** 10.3389/fspor.2026.1788596

**Published:** 2026-04-13

**Authors:** Nadim Nassif, Jessica R. El-Khoury

**Affiliations:** 1 Department of Physical Education and Sports, Notre Dame University-Louaize (NDU), Zouk Mosbeh, Lebanon; 2 Department of Media Studies, Notre Dame University-Louaize (NDU), Zouk Mosbeh, Lebanon

**Keywords:** countries, ecosystem, elite sport, leverage, media

## Abstract

Media's role in elite sport has grown exponentially from the second part of the 20th to the first part of the 21st century. It encompasses many aspects such as visibility, revenue generation, fan engagement, athlete branding, event promotion, policy decisions, international benchmarking, national pride, and cultural influence. A strong and symbiotic relationship between elite sports and the media is essential for sustained success and growth in the modern sports industry. The objective of this paper is to highlight the leverage that media has in a country's national elite sport ecosystem. This evaluation will consist of two frameworks of analysis, one that explains media's structural role in nations' success in international competitions, and another which explains its capacity to elevate sport into an instrument of power in international relations. This research identifies the unique position that media has in a country's national elite sport ecosystem and is recommended for theoretical advancement and practical application.

## Introduction

An expanding field of research investigates the complex interdependence of media and elite sport. For Gantz and Wenner (1), media coverage provides elite athletes and sports organizations an increased visibility and exposure to a wide audience. Billings and Angelini (2) believe that media outlets create compelling narratives that captivate audiences and cultivate a sense of connection with the sports they follow. For Gratton and Solberg (59), media rights deals and sponsorship agreements bring in substantial revenue which can be reinvested in sports infrastructure, training facilities, and athlete development programs. Chandler and Nauright (3) advocate that media coverage serves as a powerful promotional tool for sporting events, attracting both spectators and sponsors. For Hutchins and Rowe (60), media coverage shapes public perceptions and attitudes towards sports and athletes, influencing societal norms and values. Hutchins and Rowe (2013) also highlighted how the advent of digital and social media platforms have revolutionized how sports are consumed and followed. This accessibility allows for greater engagement from the public and can drive up interest and participation rates in various sports. For Tian et al. (4) social media has become a powerful tool for sport by exposing broader audiences to sports content, lowering discovery barriers, and pushing participation intentions. Social media platforms entertain, spread awareness, highlight norms, and even feed grassroots sport. These social media networks enable two-way relationship building among clubs, federations, and different sport events, which is essential for sport development, especially since the field has shifted from one-way promotion to service-dominate, co-create engagement, where fans, athletes and organizations jointly produce value (5, 53). This type of interactivity is a prominent tactic that assists in growing and sustaining online sport communities and by extension offline outcomes such as ticketing and memberships (6, 54). For Abeza and O'Reilly (7), social media platforms have been shown to strengthen stakeholder ties that are vital for the development of sport. For Muller et al., (8), social media metrics inform sponsorship pricing and market value proxies alongside performance indicators.

In addition, for athletes, social media reduces dependence on traditional media, allowing direct access to fans and sponsors, therefore, democratizing athlete branding and resource acquisition. For example, posts and attention to posts, like the Name-Image-Likeness created an economic venue for athletes as well as commercial opportunities beyond traditional sports revenue, allowing athletes to fund travel and training (9). With traditional media, this type of distribution was limited to specific athletes and historically constrained athlete development. However, with the advent of new media such as various social media platforms, athletes can create their own valuations. These platforms enabled visibility to a larger, even measurable audience, leading to greater sponsorship or income that can be reinvested in athletic development (10).

For Piché et al. (11), social media platforms give women's leagues and athletes direct channels to mobilize fans and sponsors, unlike traditional media with its heavy gatekeeping. This shift from selective (e.g., television) to direct with high engagement (e.g., social media) helped counter what was presumed as underexposure (12).

In their research on media and women elite athletes in the Arab world, Nassif and El-Khoury (13) have shown how the growth of media coverage in sport had an incidence on the development of the social status of elite athletes. The first objective of this paper is to point out the role that media has in a national sport system and its impact on countries' performances in elite sport. Three research-based holistic approaches will be examined for this purpose: the SPLISS (Sport Policies Leading to International Sport Success) (14), WISE (Women, Institutionalization, Specialization, and Early Learning) (15), and GGIFTE (Geography, Genetics, Interest, Funding, Transparency, Expertise) (16) models. This research will emphasize the GGIFTE framework because unlike SPLISS and WISE, which are based on Olympic Medal Table (OMT) results, GGIFTE utilizes the World Ranking of Countries in Elite Sport (WRCES), an indicator that evaluates all countries participating in international competition. The second objective is to identify media's capacity to elevate sport into an instrument of power in international relations. For this purpose, we will base our analysis on the research done by Méndez and Ruvalcaba (61) on the connection between elite sport performance and power in international relations.

### Media's position as an essential stakeholder in a country's success in elite sport

#### Overview of the SPLISS and WISE models

Created in 2003, the SPLISS consortium developed and shared expertise in elite sport policy in cooperation with policymakers, National Olympic Committees (NOCs), international sport federations (IFs), and researchers worldwide (17). Based on a comparison of 15 countries, SPLISS proposed a model structured around nine interacting pillars: (1) Financial support; (2) Governance of the elite sport system; (3) Sport participation; (4) Talent identification system; (5) Athletic career support; (6) Training facilities; (7) Coaching development; (8) National and international competitions; (9) Scientific research.

The WISE model identified four key factors that determine countries' success in international competitions: development of women sport; institutionalization of sport by the state; specialization in sports where there is a higher chance to win medals; and investment in sports newly added to the Olympic program.

For Reiche (15), women sports are subject to a lower level of competition for countries than their males' counterparts, so investing on women athletes gives more opportunities to win medals. Implementing a national sport system where athletes will have a proper structure to hone their skills is indispensable for success. In addition, the more a country specializes in specific sports, the more it increases its probability of succeeding in them. Lastly, sports that are not yet established in the Olympics do not have a high level of competition, and countries must therefore take advantage of this situation and invest in them before their competitors do (15).

However, media is not formally included in either the SPLISS or WISE models. Although both frameworks appear comprehensive, they focus mainly on meso-level factors and overlook macro-level influences such as economic and political conditions (18), which are crucial for understanding media impact. Both models also rely on the OMT indicator to assess national performance, which presents several limitations (19). First it ignores differences in sport-specific levels. Second, it covers less than half of officially recognized sports. Third, it ranks fewer than half of the countries participating in international competitions.

Addressing these limitations was the first step that Nassif and Raspaud (16) have encompassed to develop a more holistic framework named GGIFTE, which purpose was to explain about the factors leading to countries' success in elite sport. The outcome assessment they adopted (19) was based on the WRCES, a research-based index benchmarking the performance of all the National Olympic Committees (NOCs), in all the officially recognized sports (20).

#### The WRCES and what it reveals on the factors behind national sport success

The main features of the WRCES are outlined by Nassif (20). First, it uses a computation model that assigns each country an annual share of points in at least one sport. Countries are then ranked based on the total points accumulated across all sports. Second, it accounts for differences in competition levels across sports. This is done using universality and media popularity coefficients. Universality reflects the number of countries participating in a sport. Media popularity measures international media coverage and audience ratings (see Appendix). Greater media coverage attracts private and public funding and raises competition's level by engaging the most talented athletes. By taking into account the universality and media popularity of each sport, the goal of the WRCES is to give a differential weight for minor and major sports. This aspect of the WRCES elevates the WRCES above the OMT and was further scientifically proven by Nassif and Millet (21) when they conducted a correlation study between the competitive level and bibliometrics of Olympic sports. They found that there is a strong correlation between the number of scientific citations or articles and the competition level determined by the WRCES while no relationship with the number of medals available for each sport at the Olympics (21).

Since it ranks 206 countries, the WRCES allows to identify the macro-level factors behind their success in elite sport. Nassif and Raspaud (22) have measured the correlations between the points won by these 206 countries in the WRCES with their population size, Gross Domestic Product (GDP), GDP per capita and research output rankings for six consecutive years: 2014, 2015, 2016, 2017, 2018 and 2019. This study was undertaken to evaluate the impact of macro-level variables such as demography, wealth, and scientific advancement on countries' performances in elite sport. Population and wealth were considered because they were subject to extensive studies in the literature related to the factors leading to countries' success in elite sport (22, 56). The information for population size, GDP, and GDP per capita, which are respectively the number of inhabitants, the total amount of US dollars (in million), and the total amount of US dollars per capita, were retrieved from the United Nations Statistics Divisions in 2021 (58).

Research output ranking was examined because Nassif & Raspaud (22) considered that the “establishment and optimization of meso and micro factors cannot be achieved without an extensive knowledge in political science, public administration, sports management, engineering, architecture, sports medicine, sports physiology, sports psychology, biomechanics, nutrition, coaching, performance analysis, and strength and conditioning” (p.61). Since all these subjects touch the different main academic fields (natural sciences, engineering, life sciences, medical sciences, and social sciences), they have been chosen to compare the points won in the WRCES with the number of publications of each country. The research output ranking was retrieved in 2022 from the SCImago Journal & Country Rank (57). The comparison was computed using Pearson's correlation. The variables were not log-transformed and the analysis was conducted using the raw values to maintain direct interpretability. To ensure that there is no bias in the correlations' coefficients, it was verified if the linear relationship were consistent across the distribution. The data (*n* = 206) was evaluated for direction (positive or negative outcome) and magnitude in reference to the ranges listed below. The results were as seen in Table 1.

**Table 1:** Results of Nassif & Raspaud’s correlations calculi between countries' points in the WRCES, population (number of inhabitants), GDP (total amount of US dollars), GDP per capita (total amount of US dollars per capita), and research output (total number of publications) for the years 2014, 2015, 2016, 2017, 2018, and 2019 (22).

Years	Correlations	Correlations	Correlations	Correlations
	WRCES/Population	WRCES/GDP	WRCES/GDP per capita	WRCES/Research output
2014	0.39	0.78	0.23	0.82
2015	0.35	0.76	0.28	0.81
2016	0.34	0.76	0.28	0.81
2017	0.30	0.75	0.29	0.79
2018	0.30	0.75	0.30	0.78
2019	0.34	0.74	0.29	0.78

According to Lee (23), Pearson correlation is characterized by three types:

- Positive correlation, when one variable increases the other tends to increase.
- Negative correlation, when one variable increases the other tends to decrease.
- No correlation, when one variable increases, the other does not tend to either increase or decrease.

The strength of a correlation is designed as follows:

- Positive values mean positive correlation with 1 being the perfect correlation.
- Negative values mean a negative correlation with −1 being the perfect correlation.
- 0 means no correlation.

Whether positive or negative, the strength of a correlation is considered as follows:

- 0–0.19: very weak
- 0.2–0.39: weak
- 0.4–0.59: moderate
- 0.6–0.79: strong
- 0.8–1: very strong

Pearson's correlation indicated highly statistically significant results (*p* < .001). Even the weaker correlations, such as WRCES/GDP per capita reached a significance level of *p* *<* 0.001, confirming that these relationships are not due to random chance.

Outliers were addressed by inspecting scatter plots of the 206 data points. While extreme values exist (such as the GDP of the USA and China), they align with the overall upward linear trend of the data. Their presence reinforces the relationship rather than distorting it. Furthermore, the big sample size (*n* = 206) reduces the disproportionate influence of any single country on the final correlation coefficient.

As observed in these results, the correlation between the WRCES and the population is weak. This contradicts the dogmatic misconception advocated by numerous newspapers, which use the medal per capita ranking to identify the countries that can “punch above their weight” (24). Less populated countries usually top this ranking and are consequently considered the most efficient in terms of international sport results. This statement seems to be very inaccurate whether we address the Olympic Medal Table or the WRCES.

In terms of medal collection first, several scholars have advocated the low impact of the population on sport results. Bernard and Busse (25), Den Butter and Van Der Tak (26) and De Bosscher (14) provide the simplest reason behind that, explaining that no matter how populated countries are, they can only send a limited, often the same number of athletes in every event. By providing a measurement on a wider number of countries, the WRCES explains with more accuracy the low importance of population.

The strong correlation between the WRCES scores and GDP (0.74–0.78) shows that total national wealth, rather than average individual wealth, is a key factor in sporting success. In contrast, the correlation between WRCES scores and GDP per capita is weak (0.23–0.30). This finding supports research by Henry et al. (18), which concludes that elite sport success depends more on national investment consecrated to elite sports than on personal income levels or wealth. Scientific advancement shows the strongest relationship with international sport performance. Research output maintained an average correlation of 0.80 with WRCES scores over six consecutive years. This makes scientific development the most consistent predictor of success in international competitions. However, these correlations do not prove direct causation. They should be interpreted as descriptive rather than explanatory. Endogeneity may be present. For example, both strong research performance and high WRCES scores may result from broader institutional development. Therefore, these bivariate correlations describe the macro-level structure of the WRCES. They do not establish definitive structural or causal relationships.

By examining why population size and GDP per capita have limited influence, Nassif and Raspaud (27) identified key factors that shape national success in elite sport. This analysis clarified the structural role of media within national elite sport ecosystems. By highlighting first the significance of wealth and research output, they were able to show that, without financial resources and scientific know-how, essential to develop the skills of the actors of the national sports movements (administrators, coaches and athletes), a large population provides no advantage.

By exploring the other factors hindering the impact of population, Nassif and Raspaud (27) have found that geography, defined by countries' cold weathers, is a major player in winter sports. Indeed, largely populated nations such as Nigeria and Bangladesh cannot succeed in winter sports without snow on their home soil. This will systematically limit their access to a high number of sports and affect their overall performance. This structural limitation reduces their overall performance. WRCES results confirm this pattern: the top twenty countries between 2014 and 2019 all scored points in winter sports (28).

Geography was not the only natural factor reducing the impact of population size on countries' performances. For Nassif and Raspaud (27), population's average height, which they referred to as genetics, affect significantly the incidence of population in strength related sports, where weight categories were implemented to prevent taller and heavier people from having an unfair advantage. They took three examples of populated countries with low average heights, Thailand, Mexico, and China, in “weight categories sports” where they are very successful, respectively Muay Thai, boxing, and weightlifting. These three nations were among the most successful in the low weight categories but had very few athletes appearing in the heavier ones. Therefore, in sports without weight divisions displaying physical confrontation, such as football, basketball, and rugby, average height will have an even bigger impact. As a matter of fact, Nassif and Raspaud (16) conducted statistical analysis on a sample of 65 countries and found that those with a higher average size do constantly better in the WRCES.

Nassif and Raspaud (24) have also highlighted the importance of social behavior on countries' success in elite sport by showing that some societies are keener than others to participate in sports. This infatuation will enlarge mass participation and the pool of talents. Here, it is neither the size of the population nor the value of the GDP per capita that will create the conditions to develop talented athletes, but the interest that the government and/or the local communities will have in providing the adequate infrastructure (sports facilities and coaches) that will facilitate the engagement of this mass of participants in sport competitions (24). Without this propitious environment set up by the public actors, a large population and a high GDP per capita will be obsolete. Recognizing the limited role of population and GDP per capita helped define the foundations of the GGIFTE model. This framework emphasizes structural determinants of elite sport success, particularly the central role of media, discussed in the next section.

#### The GGIFTE model and the impact that media has on success in elite sport

The GGIFTE model is based on the six variables identified by Nassif and Raspaud: wealth, scientific advancement, geography, genetics, cultural, and political interest (27). Because geography and genetics are natural factors that could not be altered, Nassif and Raspaud (27) consider them to be the first pre-requisites. The latter are then followed by political and cultural interests, which will generate funding, mechanism that is highly dependent on the country's wealth. Nassif and Raspaud (27) advocated that for funding to be efficient, it must avoid corruption in its different forms (bribery, kickbacks, embezzlements of funds, clientelism, nepotism, conflicts of interest etc.), so transparency is essential for the optimal functioning of a national elite sport system. Indeed, once money is invested into the structure, transparency will create the adequate environment that will permit to the sports associations (SAs), national sport governing bodies (NSGBs), National Olympic Committees (NOCs) and national sports federations (NSFs) to develop the proper strategies to succeed. These meso- and micro-level policies could not reach their objectives without the input of the media. Indeed, public funding for elite athletes is very rare, and when it occurs, it is most of the time insufficient to allow them to train full-time for their sports. Nassif and Raspaud (29) have compiled a list of American Olympians who all had a full-time job besides their athletic careers. Those elite athletes concerned a wide range of sports' disciplines: triathlon, modern pentathlon, athletics, rowing, wrestling, diving, cycling, snowboarding, curling, shooting, Nordic combined etc. For these athletes to receive funding from endorsement deals, the SAs, NSFs, NOCs, and NSGBs must systematically look for the media, to enhance their exposure and attract agents from the private sector. This was highlighted by Herold and Breuer (30) who found that professional sports organizations generate a significant portion of their incomes from the sale of broadcasting rights and sponsorships, which are directly tied to the size of the audience the media can attract. For Yoo et al. (31), media exposure provides a platform for sponsors, who in turn will give the financial backing necessary for elite training. For Yenlimez (32), media interest will allow higher financial resources which will provide better sporting infrastructure, specialized coaching, and sports science support, all of which are essential for success in international competitions. On the other hand, when media representation is poor, it will hobble participation and funding and systematically limit performance potential. This aspect was also highlighted by Rahmani et al. (62) who found that financial support is essential for improving athletes' performances and ensuring long-term career sustainability. Stable financial backing will allow athletes to have better access to training, recovery, and mental health resources, allowing them to focus on their sporting development.

Based on the GGIFTE model, media's role is therefore central in a national elite sport structure. It is part of the “Interest” sequence of the framework, alongside the infatuation of the citizens to attend events, and the public and private sectors to invest. Media is therefore one of the major stakeholders, besides the citizens, public and private sectors that will provide funding for elite sport. It is an indispensable vector that will be able on one hand, to attract the fans, which are the citizens of a country, and on the other the private sector, which will fund the teams and athletes. Media itself is also a major financial investor in the elite sport industry. Indeed, in 2024, the global value of sports media rights exceeded 60 billion US dollars (33).

Media functions as a communication channel, a measurement system (e.g., real-time analytics), and a marketplace (e.g., visibility, branding, sponsorship). Hence, to deploy media effectively for elite athlete development, organizations must match media tactics to place, stakeholder incentives, resource flows, governance needs, and the competencies of athletes (5). These actions must consider the geographic variables which shapes audience languages, time zones, platform preferences, local media ecosystems, and political/regulatory constraints, thus a “one-size-fits-all” media strategies will not be sustainable (34). Some media considerations as actionable strategies in this regard can be implementing or producing region-specific cuts of highlights, athlete stories, and adding local language captioning. Also, these stories can embed training environments or local coaching traditions into storytelling to strengthen place-identity and attract regional funding or partners. Broadcasters, national federations, sponsors, municipal authorities and community groups each have different objectives, however coordinated media strategies multiply reach, reduce friction, and perhaps most importantly translate athlete visibility into systemic support (55). Therefore, effective use of media to support elite athletes requires place-sensitive content, stakeholder alignment, clear funding and reinvestment paths, transparent governance, upskilling of micro-participants, and real-time analytics that feed operations and welfare. After analyzing the functional role that media has in a country's sport success, we will go through the second part of this research, which consists of understanding its capacity to raise sport in an instrument of power in international relations.

#### Media's capacity to elevate sport into an instrument of power in international relations

For De Bosscher et al. (17), establishing a successful elite sport policy goes beyond the winning of medals. It is a virtuous circle where sport success will trigger national pride, international prestige, and public interest in sport. For Green and Houlihan (35), these attributes make of sport a major player in a country's diplomacy. This was further highlighted by Reiche (36) who found out that as soon as a country becomes independent, its branding as nation becomes a priority in its foreign policy, and sport success is a key component in this strategy. In this aspect, Henry et al. (37), showed how Egypt's involvement in international sport in the 1950s was a political message of a new emerging nation refusing western imperialism. Bouchet and Kaach (38) have also highlighted how newly independent African countries such as Morocco, Algeria, Tunisia, Senegal, Benin, Congo, and Cameroon have implemented national sport programs as part of their strategies to gain international political recognition.

If several research have highlighted the investment that governments have poured into sport for international political recognition, very few have accurately measured how much sport can be used as an instrument of power for countries in international relations. Méndez and Ruvalcaba (2025) were pioneer in evaluating how much performance in elite is connected to power in international relations. They have undertaken correlation calculi between the World Power Index (WPI), an indicator developed by Ruvalcaba measuring national power (39), the OMT and the World Sport Power Index (WSPI).

The WPI is composed of three categories of capacities: Material (which are tangible “hard” power assets such as GDP and military expenditure), Semi-material (consisting of indicators of developments such as infrastructure, energy production, and health), and Immaterial (“soft power”, made of qualitative influence like educational quality, media outreach, diplomatic strength, and institutional stability). The WSPI is the first research-based indicator measuring countries' capacities to use sport as a vector of national power (40). Two comparable rankings were initiated in 2025: the SKEMA Sport and Soft Power Index and the Sport Soft Power States Rankings created by the Polish Institute of Sports Diplomacy (PISD). The SKEMA Sport and Soft Power Index, an annual study produced by SKEMA Publika, the think tank of SKEMA Business School, includes criteria such as government policy, elite performance, event staging, and commercial power (41). The PISD Sport Soft Power States Rankings which considers the role of sports in foreign policies, public and private sector sport sponsorships, the presence of officials in decision-making bodies of international federations, the recognition and achievements of athletes and a country's involvement in hosting sports events (42). Unlike the WSPI, the methodologies of both the SKEMA Sport and Soft Power Index and the PISD Sport Soft Power States Rankings were not published in peer-reviewed scientific journals.

The WSPI is composed of three variables: the performance of national teams, global popularity of the local professional sport leagues, and the hosting of major sports events. For the performance of the national teams, it uses the WRCES. For the hosting of major sports events, it uses an indexed named the Ranking of Sports Cities (RSC), which was created in 2012 by the global communication agency Burson (43). The RSC uses online surveys to account for the views of IFs and sports industry experts regarding the cities' capacities to host major sports events, combined with the frequency of their association with sport on the internet. The third component, global popularity of the local professional sports leagues, is measured by calculating the number of times a professional league is cited in each country's top sport website. To avoid overlapping with the second variable of the WSPI, which calculates the hosting of major sports events, Nassif has chosen to dismiss from the RSC the association between sport and a city on the internet, and only consider the points related to the IFs and sports industry experts' surveys (40). A country's final score in the WSPI will be equal to the average of its points in the three variables explained above: the performances of national teams (through the WRCES); global media popularity of the local professional sport leagues; and hosting of major sports events (through 50% of the RSC) (40).

Méndez and Ruvalcaba (2025) have found that the correlation between the WPI and WSPI, which value exceeded 0.9, was much superior to the one between the WPI and the OMT, which stood at 0.7. And unlike the OMT, the WSPI can include all the countries having NOCs (Méndez and Ruvalcaba, 2025). This very strong correlation between the WPI and WSPI demonstrates that sport has the capacity to reveal national power. This fact was made possible due to the global outreach of the media. Indeed, the presence of media in elite sport has grown exponentially since 1960s when the Rome Summer Olympics was covered for the first time by the US network Columbia Broadcasting System (44). In the early days of sport broadcasting, there were no accurate measurement of international audience. In the 1968 Mexico City Games, it was estimated that 600 million people watched the competition. In 1984, in Los Angeles, this number grew to 900 million, and in 1992, in Barcelona to 3.5 billion (63). This number continued to rise in the 21st century reaching 4.7 billion in Beijing 2008 (45). In the last Summer Olympics to date, the one organized in Paris in 2024, the global audience reached its record, with more than 5 billion people from more than 200 countries watching the event (46). This exponential growth does not only concern the Olympics. Another mega-sport event, the FIFA World Cup, has also seen its global audience massively increasing. The first time it was broadcasted on television was the 1954 edition, organized in Switzerland. Back then, around 90 million people watched the tournament (47). In the last World Cup to date, the one organized in Qatar in 2022, this number grew to 5 billion (48). This very large audience covering more than half of the world's population shows how media became, in the first quarter of the 21st century, an indispensable vector for the global promotion of elite sport.

According to the WPI, media is a major component of a country's immaterial capacities, which Ruvalcaba defined as soft power (39). This sociological phenomenon was identified by Ziaee et al. (49), who explain how media contributed to the shaping of Iranian national identity through their participation in the 2018 FIFA World Cup and the 2019 Asian Football Confederation Cup. By broadcasting nations' achievement in elite sport, media is enabling sport to produce soft power, and therefore, strengthen national power. Media is thus the vector that will elevate sport into a solid instrument of power in international relations.

## Discussion

This paper had two objectives. The first was to show, through the GGIFTE model, a holistic framework analyzing the factors leading to countries' sport success, the structural role that media has in a national elite sport ecosystem. Media acts here as a major stakeholder providing funding and improving athletes' training and social situation. With the growth of social and digital media, this role of contributing to sport success will grow further. The massive financial opportunities that athletes get from social media had led in 2021, to have the US supreme court overrule a law that prevented college athletes in the US from being paid and allow them to earn income from their image rights (50). This breakthrough resulted, three years later, in 2024, in having universities paying directly their students-athletes (51). Media's impact on the social status of elite athletes, advocated by Nassif and El-Khoury (13), will therefore have a higher incidence in the years to come, legitimizing the position of media as an indispensable stakeholder in a national elite sport ecosystem.

The second objective of this research was to examine the impact that media has in elevating elite sport into a solid instrument of power in international relations. Here, the high correlation between the WPI, an index measuring a country's national power, and the WSPI, an indicator evaluating countries capacities to use sport for soft power, demonstrated how success in elite sport reveals national power. This process is only made possible with the global massive broadcasting of major sports competitions, allowing billions of people around the world to watch countries' achievements in sport. Media is therefore enabling sport to reveal a country's national power, and because it is a major component of states' immaterial capacities, it is driving sport into a producer of soft power, thus making of it a robust instrument of power in international relations.

Media has therefore a unique leverage in a national elite sport ecosystem. At the managerial level, it is providing a support system for athletes, and at the political level, it is acting as an indispensable channel of communication elevating sport into a significant player of a country's foreign policy. Understanding media's role open the door for practical implications for NSGB, NOCs, NSFs, SAs, and media institutions to coordinate and implement the proper media strategies, which will establish a support system for their athletes and raise the international visibility of their countries.

One of the limits of this research is that the model may be subject to reverse causality between media exposure and elite performance, which often exist in a “virtuous cycle” where each reinforces the other. This two-way relationship warrants further investigation in future research to better disentangle the direction of influence. Another area to explore is how media could be used to disempower a nation in its participation in major sports events. This was already investigated by Ziaee et al. who highlighted the negative political connotations that western media used towards the Iranian Olympic delegation in the Tokyo 2020 Olympics (52). Media can therefore be a double-edged sword that could either strengthen or weaken the international reputation of a country, and this duality must not be overlooked in future scholarly work.

This paper also only tackled the national elite sport ecosystem. It therefore opens the door for further research in sport governance as elite athletes are only a very fringe proportion of the mass of participants in competitive sport (24). It will be therefore very pertinent to undertake, in the future, scholarly work aiming at measuring the impact that media has in attracting youth and non-elite participants in engaging in sport, and thus examine its influence on the holistic sport governance of a country.

## Appendix

### Calculation of the level of competition coefficient of each sport in the WRCES

The level of competition coefficient of each sport in the WRCES will be calculated by doing the sum of its universality and popularity coefficients.

### Calculation of the universality coefficient of each sport

The universality coefficient is calculated based on the sport's number of national federations, its presence in the programs of the Olympics, the International School sport federation, International University Sports Federation, International Military Sports Council, International Police Sport Union, International Masters Games Association, World Transplant Games Federation, Special Olympics, the International Committee of Sports for the Deaf, the International Workers and Amateurs in Sports Confederation, and the Committee of the International Children's Games; all international multisport organizations recognized by the IOC in addition to having an official program.

### Calculation of the popularity coefficient of each sport

For the popularity coefficient, the frequent presence of the different sports in each country's major sport website will be measured in a one-year span. If, for example, a country has eight popular sports with a weekly appearance on the top sport website, the most popular among these sports would receive a score of 100 and the other seven sports events that are ranked below would receive points according to the rule of three, using the following calculi:

- (Points for the 2nd most popular sport * 100)/8 = (7*100)/8 = 87.5
- (Points for the 3rd most popular sport * 100)/8 = (6*100)/8 = 75.
- (Points for the 4th most popular sport * 100)/8 = (5*100)/8 = 62.5
- (Points for the 5th most popular sport * 100)/8 = (4*100)/8 = 50.
- (Points for the 6th most popular sport * 100)/8 = (3*100)/8 = 37.5
- (Points for the 7th most popular sport * 100)/8 = (2*100)/8 = 25.
- (Points for the 8th most popular sport * 100)/8 = (1*100)/8 = 12.5

These points will then be multiplied by a coefficient based on the GDP of each country. Every trillion of dollars gives one point for the GDP coefficient. The popularity points won by a sport in each country are then added to have their total number of points in the world. Since 115 sports are included in the WRCES, the most popular sport will have a first popularity sub-coefficient of 115.

All the other sports will have a first popularity sub-coefficient according to the rule of three:

(Sport A total popularity points * 115) / total popularity points of the most popular sport

The second popularity sub-coefficient will be based on the number of countries where this sport is popular. Here as well, the sport which is present in the largest number of countries will receive a second sub-coefficient of 115. The others will get a sub-coefficient based on the rule of three:

(number of countries where sport A is popular * 115) / most present sport in the world

The final popularity coefficient of each sport will be an average of the first and second sub-coefficients.

### Example of calculation of the level of competition of the sport of Handball in the 2019 WRCES edition

Handball universality coefficient + Handball popularity coefficient = Handball level of competition coefficient:

10.17 + 25.34 = 35.51

## References

[1] Gantz W. Wenner L.A. (1995). Fanship and the television sports viewing experience. Sociol Sport J, 12(1), 56–74. 10.1123/ssj.12.1.56

[2] Billings A.C. Angelini J.R. (2007). Packaging the games for viewer consumption: gender, ethnicity, and nationality in NBC’s coverage of the 2004 summer olympics. Commun Q, 55(1), 95–111. 10.1080/01463370600998731

[3] Chandler T.J. Nauright J. (2013). Making the Rugby World: Race, Gender, Commerce. London: Routledge. 10.4324/9781315036984

[4] Tian Y. Yang P. Zhang D. (2023). The relationship between media use and sports participation behavior: a meta-analysis. Digit Health, 9. 20552076231185476. 10.1177/20552076231185476 37434724 PMC10331189

[5] Filo K. Lock D. Karg A. (2015). Sport and social media research: a review. Sport Manag Rev, 18(2), 166–81. 10.1016/j.smr.2014.11.001

[6] Pronschinske M. Groza M.D. Walker M. (2012). Attracting facebook “fans”: the importance of authenticity and engagement as a social networking strategy for professional sport teams. Sport Mark Q, 21(4), 221–31. 10.1177/106169341202100405

[7] Abeza G. O’Reilly N. (2014). Social media platforms’ use in building stakeholder relationships: the case of national sport organizations. J Appl Sport Manag, 6(3), 1–23. 10.7290/jasm06ep7w

[8] Müller O. Simons A. Weinmann M. (2017). Beyond crowd judgments: data-driven estimation of market values of football players. Eur J Oper Res, 263(2), 611–24. 10.1016/j.ejor.2017.05.005

[9] Kian E.M. Zimmerman M. (2023). Social media scholarship in sport studies and college athletes’ name, image, and likeness opportunities. Int J Sport Commun, 16(3), 321–7. 10.1123/ijsc.2023-0136

[10] Stokowski S. Coor C. Rudd A. Lewis I. Powell A. Steward A. Bellamy S. Rivers J. Godfrey M. (2024). Focus on followers: an analysis of factors impacting division I athletes’ name, image, and likeness valuation. J Appl Sport Manag, 16(1), 11–20. 10.7290/jasm16wozb

[11] Piché M.C. Francksen N. Taks M. (2022). Off the court: examining social media activity and engagement in women’s professional sport: the case of the WNBA. Int J Sport Commun, 15(1), 23–48. 10.1123/ijsc.2021-0069

[12] Thomson A. Hayes M. Hanlon C. Toohey K. Taylor T. (2022). Women’s professional sport leagues: a systematic review and future directions for research. Sport Manag Rev, 26(1), 48–71. 10.1080/14413523.2022.2066391

[13] Nassif N. El-Khoury J.R. (2022). Media and women elite athletes in the Arab world: current status and perspectives for developments. Asian J Sport Hist Cult, 1(3), 271–85. 10.1080/27690148.2023.2201822

[14] De Bosscher V. (2008). The Global Sporting Arms Race: An International Comparative Study on Sports Policy Factors Leading to International Sporting Success. Aachen: Meyer & Meyer Verlag.

[15] Reiche D. (2016). Success and Failure of Countries at the Olympic Games. London: Routledge.

[16] Nassif N. Raspaud M. (2023). National Success in Elite Sport. Cham: Springer Nature Switzerland. 10.1007/978-3-031-38997-9

[17] De Bosscher V. Shibli S. Westerbeek H. Van Bottenburg M. (2015). Successful Elite Sport Policies: An International Comparison of the Sports Policy Factors Leading to International Sporting Success (SPLISS 2.0) in 15 Nations. Aachen: Meyer & Meyer Sport.

[18] Henry I. Dowling M. Ko L.M. Brown P. (2020). Challenging the new orthodoxy: a critique of SPLISS and variable-oriented approaches to comparing sporting nations. Eur Sport Manag Q, 20(4), 520–36. 10.1080/16184742.2020.1719428

[19] Nassif N. Raspaud M. (2023a). Measurement of countries’ performances and successes in elite sport: the world ranking of countries in elite sport. In: National Success in Elite Sport: Exploring the Factors That Lead to Success, 1–32. Cham: Springer Nature Switzerland. Available online at: https://link.springer.com/book/10.1007/978-3-031-38997-9_1

[20] Nassif N. (2018). World ranking of countries in elite sport. Rivista di Diritto ed Economia Dello Sport, 14(2), 55–75. Available online at: https://www.rdes.it/RDES_2_2018_NASSIF.pdf

[21] Nassif N. Millet G. (2025). For a new world ranking of countries in elite sport: correlation between competition level and bibliometrics in Olympic sports. Front Sports Act Living, 7, 1489652. 10.3389/fspor.2025.1489652 40909992 PMC12405247

[22] Nassif N. Raspaud M. (2023c). Correlations between sport results, population, GDP, area, and research rankings. In: National Success in Elite Sport: Exploring the Factors That Lead to Success, 69–75. Cham: Springer Nature Switzerland. 10.1007/978-3-031-38997-9_3

[23] Lee H., 2023. Inference of correlation and regression. In: Foundations of Applied Statistical Methods. 2nd ed. Cham: Springer, pp.99–124. 10.1007/978-3-031-42296-6_5

[24] Nassif N. Raspaud M. (2023d). Why do population and GDP per capita not have an impact on countries’ performances in elite sport? In: National Success in Elite Sport: Exploring the Factors That Lead to Success, 77–99. Cham: Springer Nature Switzerland. 10.1007/978-3-031-38997-9_4

[25] Bernard A.B. Busse M.R. (2004). Who wins the Olympic games: economic resources and medal totals. Rev Econ Stat, 86(1), 413–7. 10.1162/003465304774201824

[26] Den Butter F.A. Van Der Tak C.M. (1995). Olympic medals as an indicator of social welfare. Soc Indic Res, 35(1), 27–37. 10.1007/BF01079236

[27] Nassif N. Raspaud M. (2023e). What are the factors leading to countries’ success in elite sport and how are they related? In: National Success in Elite Sport: Exploring the Factors That Lead to Success, 101–12. Cham: Springer Nature Switzerland. 10.1007/978-3-031-38997-9_5

[28] International Center for Sport Policy and Governance. (2023). World Ranking of Countries in Elite Sport updates. Available online at: https://www.ndu.edu.lb/international-center-for-sport-policy-and-governance/home (Accessed March 20, 2026).

[29] Nassif N. Raspaud M. (2023f). Expertise of the NSGBs, NOCs, NSFs, and SAs in implementing elite sport policies. In: National Success in Elite Sport: Exploring the Factors That Lead to Success, 113–44. Cham: Springer Nature Switzerland. 10.1007/978-3-031-38997-9_6

[30] Herold E. Breuer C. (2023). Does the game matter? Analyzing sponsorship effectiveness and message personalization in sport live broadcasts. J Sport Manag, 37(4), 290–301. 10.1123/jsm.2022-0206

[31] Yoo E. Park J.H. Lee J.M. (2021). The experience and meaning of media to non-mainstream athletes: qualitative study. Sustainability, 13(3), 1154. 10.3390/su13031154

[32] Yenilmez M.I. (2021). Gender inequality and female sports participation in Turkey. Cent Eur J Sport Sci Med, 33(1), 27–41. 10.18276/cej.2021.1-03

[33] Hamilton G. (2024). Global Value of Sports Media Rights Tops $60bn. London: SportBusiness. Available online at: https://www.sportbusiness.com/news/global-value-of-sports-media-rights-tops-60bn/ (Accessed March 20, 2026).

[34] Ryser J. (2025). Why We Don’t Believe in ‘One Size Fits All’ Marketing Strategies. Go Revity. Available online at: https://gorevity.com/why-we-dont-believe-in-one-size-fits-all-marketing-strategies/ (Accessed March 20, 2026).

[35] Green M. Houlihan B. (2005). Elite Sport Development: Policy Learning and Political Priorities. London: Routledge. 10.4324/9780203022245

[36] Reiche D. (2015). Investing in sporting success as a domestic and foreign policy tool: the case of Qatar. Int J Sport Policy Polit, 7(4), 489–504. 10.1080/19406940.2014.966135

[37] Henry I.P. Amara M. Al-Tauqi M. (2003). Sport, Arab nationalism and the Pan-Arab games. Int Rev Sociol Sport, 38(3), 295–310. 10.1177/10126902030383003

[38] Bouchet P. Kaach M. (2004). Existe-t-il un « modèle sportif » dans les pays Africains francophones? Staps, 65(3), 7–26. 10.3917/sta.065.0007

[39] Morales Ruvalcaba D. (2020). The semiperipheral states in the twenty-first century: measuring the structural position of regional powers and secondary regional states. Int Stud, 57(1), 20–50. 10.1177/0020881719885532

[40] Nassif N. (2022). The world sport power index: measuring states’ capacities to use sport as an instrument of soft power. Siyasat Arabiya, 10(57), 46–57. Available online at: https://siyasatarabiya.dohainstitute.org/en/issue057/Pages/art04.aspx (Accessed March 20, 2026).

[41] Chadwick S. Widdop P. Revel C. Vidal F. Scull S. Le Bris M. (2025). Sport and Soft Power Ranking. SKEMA Publika. Available online at: https://publika.skema.edu/sport-soft-power-ranking/ (Accessed March 20, 2026).

[42] Banaziak M. Banasiewicz B. Iwanczyk P. Kobierecki M. Rajkiewicz M. (2026). Sport Soft Power 2026 States Ranking. Polish Institute of Sports Diplomacy. Available online at: https://pids.pl/raport/sport-soft-power-2026-states-ranking/ (Accessed March 20, 2026).

[43] Burson Global (2026). Official Website. Available online at: https://www.bursonglobal.com/ (Accessed March 20, 2026).

[44] De Moragas Spa M. Rivenburgh N. K. Larson J. F. (1996). Television in the Olympics. London: J. Libbey.

[45] International Olympic Committee (IOC). (2016). Olympic Marketing Fact File. Available online at: https://www.commoncorediva.com/wp-content/uploads/2016/07/olympic-marketing-fact-file-2016.pdf (Accessed March 20, 2026).

[46] International Olympic Committee (IOC). (2024). Paris 2024 Audience & Insights Report. Available online at: https://stillmed.olympics.com/media/Documents/News/2024/12/Paris-2024-Audience-and-Consumer-Insights-Report.pdf (Accessed March 20, 2026).

[47] Fédération Internationale de Football Association. (2018). 54 Days to Go: Beamed Around the World. Available online at: https://inside.fifa.com/tournaments/mens/worldcup/2018russia/news/april-21-wc-countdown-54-days-to-go-2937964 (Accessed March 20, 2026).

[48] Fédération Internationale de Football Association. (2022). FIFA World Cup Qatar 2022: Global engagement & Audience Report. Available online at: https://inside.fifa.com/tournament-organisation/audience-reports/qatar-2022 (Accessed March 20, 2026).

[49] Ziaee A. Adib-Moghaddam A. Elling A. Van Sterkenburg J. Van Hilvoorde I. (2021). Football and the media construction of Iranian national identity during the FIFA world cup 2018 and AFC Asian cup 2019. Soccer Soc, 22(6), 613–25. 10.1080/14660970.2021.1952691

[50] CNBC. (2021). Supreme Court NCAA Decision: How College Athletes Plan to Cash in. Consumer News and Business Channel. Available online at: https://www.cnbc.com/2021/06/21/supreme-court-ncaa-decision-how-college-athletes-plan-to-cash-in.html (Accessed March 20, 2026).

[51] Witz B. Shimabukuro M. (2024). Big Money. College Athletes and the N.C.A.A.: A Timeline. The New York Times. (2024). Available online at: https://www.nytimes.com/2024/05/29/us/ncaa-college-athletes-pay-history.html (Accessed March 20, 2026).

[52] Ziaee A. Lee J.S. Sterkenburg J.V. Hilvoorde I.V., 2024. Media and representation of others. Case of Iran in the 2020 Tokyo olympics. Eur J Sport Soc, 21(4), 393–410. 10.1080/16138171.2024.2345950

[53] Abeza G. (2023). Social media and sport studies (2014–2023): a critical review. Int J Sport Commun, 16(3), 251–61. 10.1123/ijsc.2023-0182

[54] Eagleman A.N. (2013). Acceptance, motivations, and usage of social media as a marketing communications tool among employees of sport national governing bodies. Sport Manag Rev, 16(4), 488–97. 10.1016/j.smr.2013.03.004

[55] Maderer D. Parganas P. Anagnostopoulos C. (2018). Brand-image communication through social media: the case of European professional football clubs. Int J Sport Commun, 11(3), 319–38. 10.1123/ijsc.2018-0086

[56] Nassif N. Raspaud M. (2023b). Literature review on the analysis of the factors behind countries’ success in elite sport. In: National Success in Elite Sport: Exploring the Factors That Lead to Success, 33–67. Cham: Springer Nature Switzerland. 10.1007/978-3-031-38997-9_2

[57] Scimago Journal & Country Rank. (2022). Scimago Portal. Available online at: https://www.scimagojr.com/ (Accessed March 20, 2026).

[58] United Nations Statistics Division. (2025). UN Data Portal. Available online at: https://unstats.un.org/UNSDWebsite/ (Accessed March 20, 2026).

[59] Gratton C Solberg HA. (2007). The economics of sports broadcasting rights. Rivista di diritto ed economia dello sport. 3(3), 11–26.

[60] Hutchins B Rowe D. (2013). Sport beyond television: the internet, digital media and the rise of networked media sport. New York, NY: Routledge.

[61] Méndez CP Morales RD. (2025). Relaciones internacionales y rendimiento deportivo: Un análisis del medallero olímpico desde el Índice de Poder Mundial [International relations and sports performance: An analysis of the Olympic medal table from the World Power Index]. Movimento*.* 31, e31030.

[62] Rahmani M Majedi N Nasab MN. (2024). The importance of financial support for professional athletes in competitive sports. Technol. Anal. Strateg. Manag*.* 3(3), 70–87. 10.61838/kman.jtesm.3.3.8

[63] Tomlinson A. (2005) Sport and leisure cultures. Minneapolis: University of Minnesota Press.

